# A novel stratification framework for predicting outcome in patients with prostate cancer

**DOI:** 10.1038/s41416-020-0799-5

**Published:** 2020-03-20

**Authors:** Bogdan-Alexandru Luca, Vincent Moulton, Christopher Ellis, Dylan R. Edwards, Colin Campbell, Rosalin A. Cooper, Jeremy Clark, Daniel S. Brewer, Colin S. Cooper

**Affiliations:** 1 Norwich Medical School, University of East Anglia, Norwich Research Park, Norwich, Norfolk UK; 2 School of Computing Sciences, University of East Anglia, Norwich Research Park, Norwich, Norfolk UK; 3 Intelligent Systems Laboratory, University of Bristol, Bristol, UK; 4 Department of Pathology, University Hospital Southampton NHS Foundation Trust, Southampton, UK; 5 The Earlham Institute, Norwich Research Park, Norwich, Norfolk UK

**Keywords:** Prostate cancer, Molecular medicine, Computer science

## Abstract

**Background:**

Unsupervised learning methods, such as Hierarchical Cluster Analysis, are commonly used for the analysis of genomic platform data. Unfortunately, such approaches ignore the well-documented heterogeneous composition of prostate cancer samples. Our aim is to use more sophisticated analytical approaches to deconvolute the structure of prostate cancer transcriptome data, providing novel clinically actionable information for this disease.

**Methods:**

We apply an unsupervised model called Latent Process Decomposition (LPD), which can handle heterogeneity within individual cancer samples, to genome-wide expression data from eight prostate cancer clinical series, including 1,785 malignant samples with the clinical endpoints of PSA failure and metastasis.

**Results:**

We show that PSA failure is correlated with the level of an expression signature called DESNT (HR = 1.52, 95% CI = [1.36, 1.7], *P* = 9.0 × 10^−14^, Cox model), and that patients with a majority DESNT signature have an increased metastatic risk (*X*^2^ test, *P* = 0.0017, and *P* = 0.0019). In addition, we develop a stratification framework that incorporates DESNT and identifies three novel molecular subtypes of prostate cancer.

**Conclusions:**

These results highlight the importance of using more complex approaches for the analysis of genomic data, may assist drug targeting, and have allowed the construction of a nomogram combining DESNT with other clinical factors for use in clinical management.

## Background

Driven by technological advances and decreased costs, a plethora of genomic datasets now exists. This is illustrated by the availability of expression data from over 1.3 million samples from the Gene Expression Omnibus,^1^ and DNA sequence data on 25,000 cases from the International Cancer Genome Consortium.^2^ Such datasets have been used as the raw material for the discovery of disease subclasses, using a variety of mathematical approaches. Hierarchical clustering, k-means clustering, and self-organising maps have been applied to expression datasets, leading, for example, to the discovery of five molecular breast cancer types (Basal, Luminal A, Luminal B, ERBB2-overexpressing and Normal-like).^3^ The inherent shortcoming of this type of approach is the implicit assumption of sample assignment to a particular cluster or group. Such analyses are in complete contrast to the well-documented heterogeneous composition of most individual cancer samples.^4,5^

Unsupervised analysis of prostate cancer transcriptome profiles using the above approaches has failed to identify robust disease categories that have distinct clinical outcomes.^6,7^ Noting that prostate cancer samples used in genome-wide studies frequently harbour multiple cancer lineages, and can have intra-tumour variations in genetic compositions,^8–10^ we applied an unsupervised learning method called latent process decomposition (LPD)^11^ that can take into account the issue of heterogeneity of composition within individual cancer samples. By heterogeneity, we mean that an individual cancer sample can be made up of several different components that each has distinct properties. We had previously used Latent Process Decomposition: (i) to confirm the presence of the basal and ERBB2-overexpressing subtypes in breast cancer transcriptome datasets;^12^ (ii) to demonstrate that data from the MammaPrint breast cancer recurrence assay would be optimally analysed using four separate prognostic categories;^12^ and (iii) to show that patients with advanced prostate cancer can be stratified into two clinically distinct categories based on expression profiles in blood.^13^ LPD (closely related to Latent Dirichlet Allocation) is a mixed membership model in which the expression profile for a single cancer is represented as a combination of the underlying latent (hidden) signatures. Each latent signature has a representative gene expression pattern. A given sample can be represented over a number of these underlying functional states, or just one such state. The appropriate number of signatures to use is determined using the LPD algorithm by maximising the probability of the model, given the data.

The application of LPD to prostate cancer transcriptome datasets led to the discovery of an expression pattern, called DESNT, that was observed in all datasets examined.^14^ Cancer samples were assigned as DESNT when this pattern was more common than any other signature, and this designation was associated with poor outcomes independently of other clinical parameters, including Gleason, clinical stage and PSA. In this paper, we test whether the presence of even a small proportion of the DESNT cancer signature confers poor outcome, and uses LPD to develop a new prostate cancer stratification framework.

## Methods

### Transcriptome datasets

Eight publicly available transcriptome microarray datasets derived from prostatectomy samples from men with prostate cancer were used, and are referred to as Memorial Sloan Kettering Cancer Centre (MSKCC),^7^ CancerMap,^14^ CamCap,^6^ Stephenson,^15^ TCGA,^16^ Klein,^17^ Erho^18^ and Karnes.^19^ There were 1785 samples from primary malignant tissue, and 173 from normal tissue (Table 1). MSKCC also had data from 19 metastatic cancer samples. The CamCap dataset was produced by combining two Illumina HumanHT-12 V4.0 expression beadchip datasets (GEO: GSE70768 and GSE70769) obtained from two prostatectomy series (Cambridge and Stockholm).^6^ The original CamCap^6^ and CancerMap^14^ datasets have 40 patients in common, and thus 20 of the common samples were excluded at random from each dataset. Each Affymetrix Exon microarray dataset was normalised using the RMA algorithm,^20^ implemented in the Affymetrix Expression Console software. For CamCap and Stephenson, previous normalised values were used. For the TCGA dataset, the counts per gene, previously calculated, were used^16^ and transformed, using the variance-stabilising transformation implemented in the DESeq2 package.^21^ For the CamCap and CancerMap datasets, the *ERG* gene alterations had been scored by fluorescence in situ hybridisation.^6,14^ Only probes corresponding to genes measured by all platforms were retained. The ComBat algorithm from the sva R package and quantile transformation, was used to mitigate series-specific effects. Flow diagrams presenting each of the analyses performed in this study, with the datasets used, are shown in the Supplementary Materials. The ethical approvals obtained for each dataset are listed in the original publications.

**Table 1:** Transcriptome datasets.

Dataset	Primary	Normal	Type	Platform	Citation
MSKCC^7^	131	29	FF	Affymetrix Exon 1.0 ST v2	Taylor et al.^7^
CancerMap^14^	137	17	FF	Affymetrix Exon 1.0 ST v2	Luca et al. 2017
Stephenson^15^	78	11	FF	Affymetrix U133A	Stephenson et al.^15^
Klein^17^	182	0	FFPE	Affymetrix Exon 1.0 ST v2	Klein et al.^17^
CamCap^6^	147	73	FF	Illumina HT12 v4.0 BeadChip	Ross-Adams et al.^6^
TCGA^16^	333	43	FF	Illumina HiSeq 2000 RNA-Seq v2	TCGA network 2015
Erho^18^	545	0	FFPE	Affymetrix Exon 1.0 ST v2	Erho et al.^18^
Karnes^19^	232	0	FFPE	Affymetrix Exon 1.0 ST v2	Karnes et al.^19^

The MSKCC study additionally reported expression profiles from 19 metastatic cancers. The ethical approvals obtained for each dataset are listed in the original publications.

### Latent process decomposition

LPD^11,12^ is an unsupervised Bayesian approach that breaks down (decomposes) each sample into component sub-elements (signatures). Each signature is a representative gene expression pattern. LPD is able to classify complex data based on the relative representation of these signatures in each sample. LPD can objectively assess the most likely number of signatures. We assessed the hold-out validation log-likelihood of the data computed at various numbers of signatures, and used a combination of both the uniform (equivalent to a maximum likelihood approach) and non-uniform (missed approach point) priors to choose the number of signatures. For input, each dataset was reduced to probes that detect the 500 genes with the greatest variance across the MSKCC dataset. For robustness, LPD is run 100 times with different seeds, for each dataset. Out of the 100 runs, we selected the run with the survival log-rank *p*-value closest to the mode as a representative run that was used for subsequent analysis.

### OAS-LPD

The OAS-LPD (one added sample-LPD) algorithm is a modified version of the LPD algorithm in which new sample(s) are decomposed into LPD signatures, without retraining the model (i.e. without re-estimating the model parameters *µ*_gk_, *σ*^2^_gk_ and *α* in Rogers et al.^11^). Only the variational parameters *Q*_kga_ and *γ*_ak_, corresponding to the new sample(s), are iteratively updated until convergence, according to Eq. (6) and (7) from Rogers et al.^11^ LPD as presented by Rogers et al.^11^ was first applied to the MSKCC dataset of 131 cancer and 29 normal samples, as described above. The model parameters *µ*_gk_, *σ*^2^_gk_ and *α*, corresponding to the representative LPD run, were then used to classify additional expression profiles from all datasets, one sample at a time. A detailed description is provided in the Supplementary Methods.

### Statistical tests

All statistical tests were performed in R version 3.3.1. For characterisation of signatures, each sample was assigned to the signature that had the largest gamma (*γ*) value for that sample.

### Correlations

Pearson correlations between the expression profiles between the MSKCC and CancerMap were calculated for each of the eight signatures: (i) for each gene, we select one corresponding probe at random; (ii) for each probe, we transformed its distribution across all samples to a standard normal distribution; (iii) the mean expression for each gene across the samples assigned to signature *j* (gene subgroup mean) in each dataset was determined; (iv) the Pearson’s correlation between the gene subgroup mean expression profile in MSKCC vs the gene subgroup mean expression profile in CancerMap is calculated for each signature.

### Differentially expressed and methylated features

Differentially expressed probe sets were identified for each signature by using a moderated *t* test implemented in the limma R package (Benjamin–Hochberg false discovery rate <0.05, differentially expressed in at least 50/100 runs; samples assigned to the signature vs the rest).

Differential methylation was assigned at the probe level. Hypo- and hypermethylated genes that are predictive of transcription were identified using the methylMix R package (functionally differentially methylated in at least 50/100 runs), using genes that are found to be differentially expressed in that signature as input. Datasets where there were <10 samples assigned to a signature were removed from the identification of intersection genes for that signature.

### Survival analyses and nomogram

Survival analyses were performed using Cox proportional hazard models, the log-rank test and Kaplan–Meier estimator, with biochemical recurrence after prostatectomy as the end point. For nomogram construction, the Cox proportional hazard model was fitted on the meta-dataset obtained by combining MSKCC, CancerMap and Stephenson datasets, and validated on CamCap, using the rms R package. The Gleason grade was divided into <7, 3 + 4, 4 + 3 and >7, the pathological stage in T1–T2 vs T3–T4, while DESNT percentage and PSA were considered continuous covariates. The missing values for the predictors were imputed using the flexible additive models with predictive mean matching, implemented in the Hmisc R package. The linearity of the continuous covariates was assessed using the Martingale residuals.^22^ The lack of collinearity between covariates was determined by calculating the variance inflation factors (VIF) (VIF values between 1.04 and 3.01).^23^ All covariates met the Cox proportional hazard assumption, as determined by the Schoenfeld residuals. The internal validation and calibration of the Cox model were performed by bootstrapping the training dataset 1000 times. The calibration of the model was estimated by comparing the predicted and observed survival probabilities at 5 years. For comparing the discrimination accuracy of two non-nested Cox models, the U-statistic calculated by the Hmisc rcorrp.cens function was used.

### Detecting over-representation of genomic features

Mutated cancer genes identified by the Cancer Genome Atlas Research Network (2015)^16^ were examined at the sample level. The under-/over-representation of these features in samples assigned to a particular LPD signature was determined using the *χ*^2^ independence test.

### Pathway over-representation analysis and signature correlation analysis

The GO biological process annotations were tested for over-representation (or under-representation) in the lists of differentially expressed genes in each signature, using clusterProfiler version 3.4.4. For a given pathway and a given sample, the pathway activation score was calculated as indicated in Levine et al.^24^ Using the complete combined dataset of all eight datasets, *Z* scores were calculated for each sample for each of the 17,697 MSigDB v6.0 gene sets. These were correlated with DESNT *γ* values, and the top 20 sets with the highest absolute Pearson’s correlation were selected. The resulting *p* values from pathway over-representation analysis were adjusted for multiple testing using the false discovery rate.

## Results

### Presence of DESNT signature as a continuous variable is associated with poor clinical outcome

**Fig. 1:**
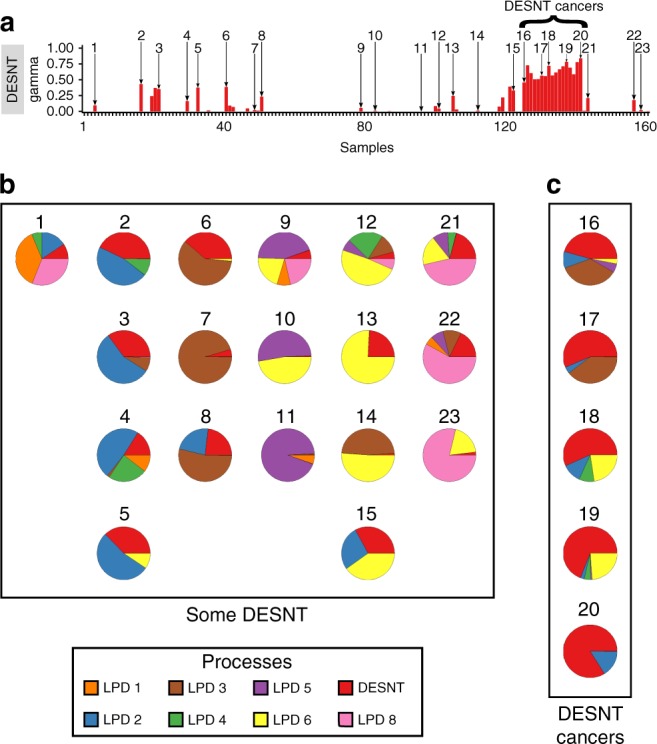
LPD decomposition of the MSKCC dataset. **a** DESNT bar chart from the LPD decomposition of the MSKCC dataset,^14^ showing the number ID assigned to 23 example samples that had some amount of DESNT signature. **b**, **c** Pie charts showing the relative proportions of the eight LPD signatures in 23 example samples. DESNT is in red; other LPD signatures are represented by different colours as indicated in the key. The number next to each pie chart indicates which cancer it represents from the bar chart above. Individual cancer samples were assigned as a “DESNT cancer” when the DESNT signature was the most abundant; examples are shown in the right-hand box (**c**, “DESNT”). Many other cancer samples contained a smaller proportion of DESNT cancer: examples shown in a larger box (**b**, “SOME DESNT”).

In our previous studies, LPD was detected between three and eight underlying signatures (also called processes) in expression microarray datasets collected from prostate cancer samples after prostatectomy.^14^ Decomposition of the MSKCC dataset^7^ gave eight signatures.^14^ Figure 1a illustrates the proportion of the DESNT expression signature identified in each MSKCC sample, with individual cancer samples being assigned as a “DESNT cancer” when the DESNT signature was the most abundant as shown in Fig. 1a and Fig. 1c. Based on PSA failure, patients with DESNT cancer always exhibited poorer outcome relative to other cancer samples in the same dataset.^14^ The implication is that it is the presence of regions of cancer containing the DESNT signature that conferred poor outcome. If this idea is correct, we would predict that cancer samples containing a smaller contribution of DESNT signature, such as those shown in Fig. 1b for the MSKCC dataset, should also exhibit poorer outcome.

**Fig. 2:**
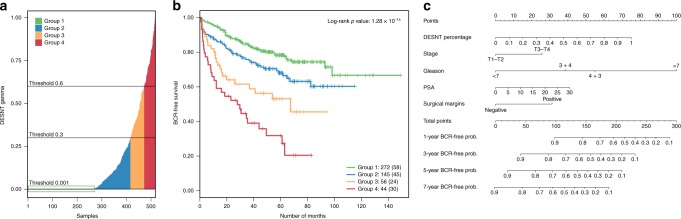
Stratification of prostate cancer samples based on the percentage of DESNT cancer present. For these analyses, the data from the MSKCC, CancerMap, CamCap and Stephenson datasets were combined (*n* = 503). **a** Plot showing the proportion of DESNT signature in each cancer sample, and the division into four groups of increasing DESNT. Group 1 samples have a proportion of <0.001 of the DESNT signature. **b** Kaplan–Meier plot showing the biochemical recurrence (BCR)-free survival based on the proportion of DESNT cancer present, as determined by LPD. The number of cancer patients in each group are indicated (bottom right), and the number of PCR failures in each group are shown in parentheses. The definition of Groups 1–4 is shown in Fig. 2a. Cancer samples with proportions up to 0.3 DESNT (Group 2) exhibited poorer clinical outcome (*χ*^2^ test, *P* = 0.011) compared with cancer samples lacking DESNT (<0.001). Cancer samples with the intermediate (0.3–0.6) and high (>0.6) proportions of DESNT also exhibited significantly worse outcome (*P* = 2.6 × 10^−5^ and *P* = 8.3 × 10^−9^, respectively, compared with cancer samples lacking DESNT. The combined log-rank *P* = 1.3 × 10^–8^). **c** Nomogram model developed to predict PSA-free survival at 1, 3, 5 and 7 years using proportion of DESNT. Assessing each clinical variable in a single patient has a corresponding point score (top scales). The point scores for each variable are added to produce a total point score for each patient. The predicted probability of PSA-free survival at 1, 3, 5 and 7 years, can be determined by drawing a vertical line from the total point score to the probability scales below.

To increase the power to test this prediction, we combined transcriptome data from the MSKCC,^7^ CancerMap,^14^ Stephenson^15^ and CamCap^6^ studies (*n* = 503). There was a significant association with PSA recurrence when the proportion of expression assigned to the DESNT signature was treated as a continuous variable (HR = 1.52, 95% CI = [1.36, 1.7], *P* = 9.0 × 10^−14^, Cox proportional hazard regression model). The outcome became worse as the proportion of DESNT signature increased. For illustrative purposes, cancer samples were divided into four groups based on the proportion of DESNT, with 47.4% of cancer samples containing at least some DESNT cancer (proportion > 0.001, Fig. 2a). PSA failure-free survival at 60 months is 82.5%, 67.4%, 59.5% and 44.9% for the proportion of DESNT signature being: <0.001; 0.001 to 0.3; 0.3–0.6; and >0.6, respectively (Fig. 2b).

### Nomogram for DESNT predicting PSA failure

The proportion of DESNT cancer was combined with other clinical variables (Gleason grade, PSA levels, pathological stage and the surgical margin status) in a Cox proportional hazard model, and fitted to a combined dataset of 318 cancer samples (MSKCC, CancerMap and Stephenson); CamCap cancer samples (*n* = 185) were used for external validation. The proportion of DESNT was an independent predictor of worse clinical outcome (HR = 1.33, 95% CI = [1.14, 1.56], *P* = 3.0 × 10^−4^), along with Gleason grade=4 + 3 (HR = 2.43, 95% CI = [1.10, 5.37], *P* = 2.7 × 10^−2^), Gleason grade>7 (HR = 5.05, 95% CI = [2.35, 10.89], *P* < 1 × 10^−4^) and positive surgical margins (HR = 1.65, 95% CI = [1.07, 2.56], *P* = 2.2 × 10^−2^) (Fig. S1: Supplementary Fig. 1). PSA level and pathological stage were below the threshold of statistical significance (*P* = 0.09, HR = 1.14, 95% CI = [0.97, 1.34]) and (*P* = 0.055, HR = 1.51, 95% CI = [0.99, 2.31]), respectively. At internal validation, the Cox model obtained a 1000 bootstrap-corrected C index of 0.747, and at external validation a C index of 0.795. Using this model, a nomogram was constructed for use of DESNT cancer information in conjunction with clinical variables to predict the risk of biochemical recurrence at 1, 3, 5 and 7 years following prostatectomy (Fig. 2c, Fig. S1).

### LPD algorithm for detecting the presence of DESNT cancer in individual samples

The ability of LPD to detect structure is likely to be dependent on sample size, cohort composition, disease severity range and data quality. We observed optimal decompositions varying between three and eight underlying signatures in different datasets.^14^ When we examined the two datasets that had an optimal eight underlying signatures (MSKCC and CancerMap), we noted a striking relationship: based on correlations of expression profiles, all eight of the LPD signatures appeared to be common (Fig. S2; *R*^2^ > 0.5). To provide a more consistent classification framework where the number of classes did not vary between datasets, we therefore used the MSKCC dataset and its decomposition into eight distinct signatures as a reference for identifying categories of prostate cancer type.

LPD is a computer-intensive procedure, and analyses can take days to run on a high-performance computing cluster. This would restrict ease of DESNT detection for clinical implementation. We therefore developed a variant of LPD called OAS-LPD, where data from a single additional cancer sample could be decomposed into signatures, following normalisation, without repeating the entire LPD procedure. LPD model parameters^11^ were first derived by decomposition of the MSKCC dataset into eight signatures. These signature parameters were then used as a framework for decomposition of additional data from single samples, selected in this case from a dataset, or in the future from a patient undergoing assessment in the clinic. To test this procedure, we applied OAS-LPD individually to cancer samples from MSKCC, CancerMap, Stephenson and CamCap (Fig. S3), and repeated Cox regression analysis and nomogram construction. The proportion of DESNT (*P* = 0.0011, HR = 1.53, 95% CI = [1.19, 1.98]), Gleason = 4 + 3 (*P* = 0.0061, HR = 2.83, 95% CI = [1.35, 5.96]), Gleason>7 (*P* < 1 × 10^−4^, HR = 5.39, 95% CI = [2.54, 11.44]) and surgical margin status (*P* = 0.0015, HR = 2.00, 95% CI = [1.30, 3.07]) remained independent predictors of clinical outcome (Fig. S4). Notably, the performance of the Cox model (internal validation C index = 0.742; external validation C index = 0.786) was not significantly different to that of the original separate dataset Cox model (train dataset *Z* = −0.65, two-tailed *P* = 0.52; validation dataset Z = 0.89, two-tailed *P* = 0.38; *U*-statistic), and the nomogram (Fig. S5) had almost an identical presentation of parameters to that shown in Fig. 2c. This observation is consistent with the high degree of correlation between LPD and OAS-LPD DESNT gamma values across the MSKCC, CancerMap, Stephenson and CamCap datasets (*P* = 2.39 × 10^−110^).

### New categories of prostate cancer

**Fig. 3:**
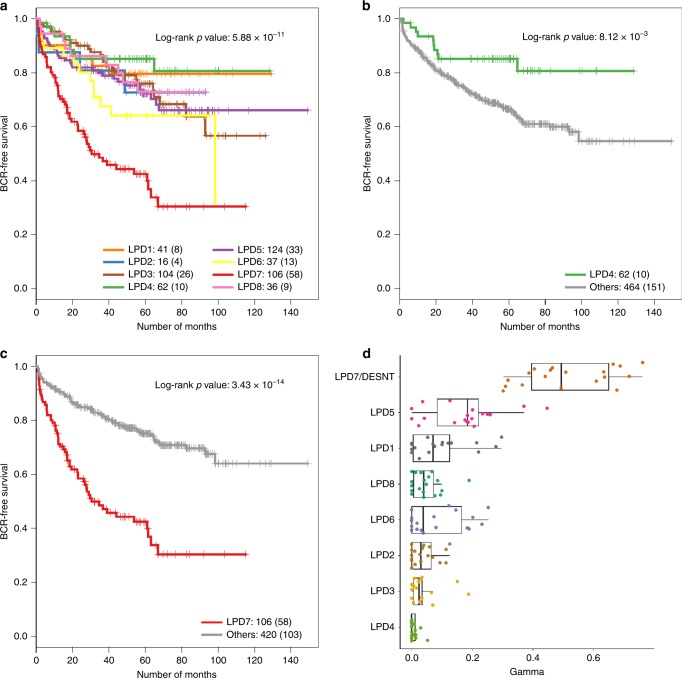
Prediction of clinical outcome according to the OAS-LPD group. **a**–**c** Kaplan–Meier plots showing PSA-free survival outcomes for the cancer patients assigned to LPD groups in analyses of the combined MSKCC, CancerMap, CamCap and Stephenson datasets: **a** comparison of all LPD groups (LPD7 is DESNT). **b** Cancer patients assigned to LPD4 compared with patients assigned to all other LPD groups. **c** Cancer patients assigned to DESNT (LPD7) compared with cancers assigned to all other LPD groups. **d** OAS-LPD signature assignment proportions for the 19 metastatic tissue samples reported as part of the MSKCC dataset. In all cases, DESNT (LPD7) was the dominant expression signature detected.

**Fig. 4:**
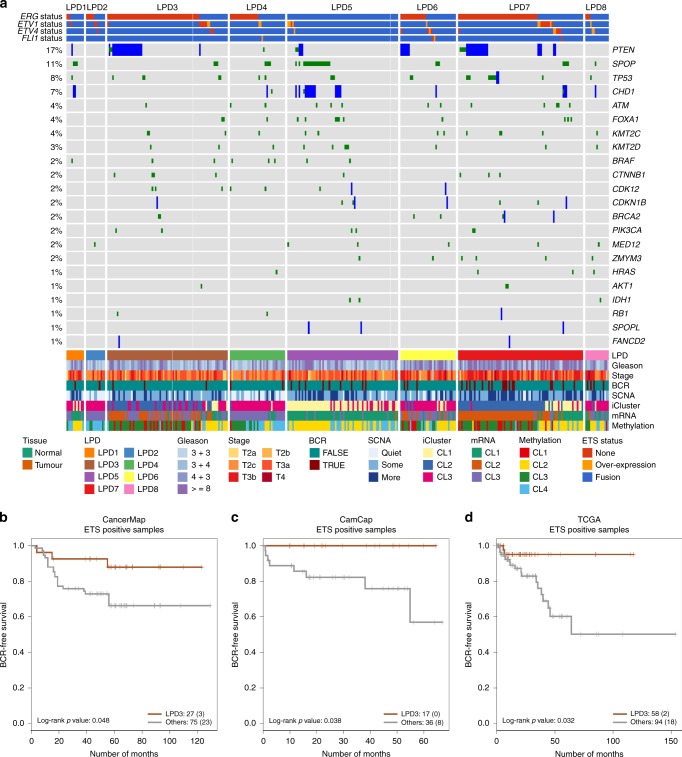
Genomic and Clinical Properies of LPD Categories. **a** OAS-LPD subgroups in The Cancer Genome Atlas Dataset (*n* = 333). Cancer samples were assigned to subgroups based on the most prominent signature as detected by OAS-LPD. The types of genetic alteration are shown for each gene (mutations, fusions, deletions and overexpression). Clinical parameters, including biochemical recurrence (BCR), are represented at the bottom, together with groups for iCluster, methylation, somatic copy number alteration (SVNA) and messenger RNA (mRNA).^16^ Comparison of the frequency of genetic alterations present in each subgroup are shown in Table 2. **b**–**d** Kaplan–Meier plots showing PSA-free survival outcomes for ETS-rearrangement positive cancers in LPD3 compared with all other ETS-positive cancers for the CancerMap, CamCap and TCGA datasets.

We wished to determine whether LPD signatures were characterised by particular clinical or molecular features, indicating that they represented distinct categories of prostate cancer. OAS-LPD using the MSKCC-derived model of gene signatures was applied to all datasets (*n* = 1958, Table 1), and each sample was assigned to the signature that was the most abundant. Samples from non-cancerous (benign) prostate tissue were more frequently assigned to LPD2, LPD4 and LPD8 than to the other groups (*P* < 0.05, *χ*^2^ test, Fig. S3, Table S1). When datasets with linked clinical data were combined (MSKCC, CancerMap, Stephenson and CamCap, Fig. 3a–c), primary cancers assigned to DESNT had worse outcome (*P* = 3.4 × 10^−14^, log-rank test, DESNT-assigned samples vs the rest), while those assigned to LPD4 had improved outcome (*P* = 0.0081, log-rank test, LPD4-assigned samples vs the rest) as judged by PSA failure. Cancer samples with *ERG* alterations assigned to signature LPD3 also exhibited better outcome (P < 0.05, log-rank test, comparison to all other ETS-positive cancer samples) in all three datasets where *ERG* status was available (Fig. 4b–d).

**Fig. 5:**
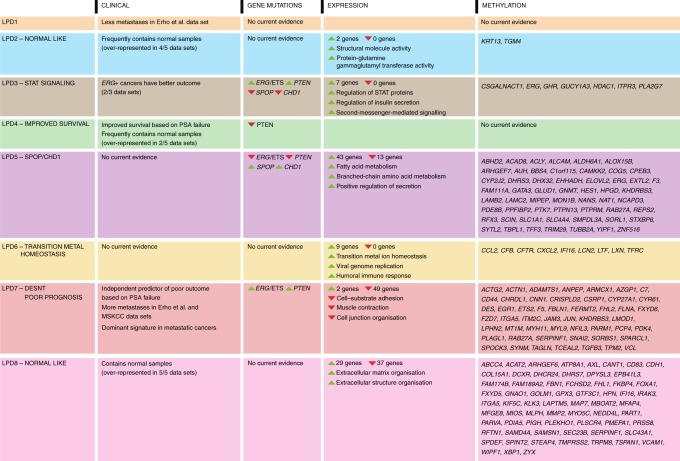
A classification framework for prostate cancer. Based on the analyses of genetic and clinical correlations, we consider that there is good evidence for the existence of LPD3, LPD4 and LPD5 as separate cancer categories, moderate evidence of the existence of LPD6 and LPD8 (based on alteration of expression only) and weak evidence for LPD1. The methylation column lists all genes that exhibit differential expression, and that also contain at least one locus that is differentially methylated.

To gain information about the new LPD categories, we examined the distribution of genetic alterations in the decomposition of the TGCA dataset^16^ (Fig. 4a). LPD3 cancer samples had over-representation of ETS and *PTEN* gene alterations, and under-representation of *CDH1* and *SPOP* gene alterations (*P* < 0.05, *χ*^2^ test, Table 2). LPD5 cancer samples exhibited exactly the reverse pattern of genetic alteration: there was under-repression of ETS and *PTEN* gene alterations, and over-representation of *SPOP* and *CHD1* alterations (Table 2). The statistically different distribution of ETS-gene alterations in samples assigned to LPD3 and LPD5, observed in the TGCA dataset, was confirmed in the CamCap and CancerMap dataset (Table 2). In summary, we have identified three additional prostate cancer categories that have altered genetic and/or clinical associations: LPD3, LPD4 and LPD5 (Fig. 5), and that may be relevant for drug targeting.

**Table 2:** Correlation of OAS-LPD subgroups with genetic alterations in The Cancer Genome Atlas Dataset.

	TCGA			CancerMap			CamCap		
	ETS–	ETS+	*χ*^2^ *P*-val	*ERG*–	*ERG*+	*χ*^2^ *P*-val	*ERG*–	*ERG*+	*χ*^2^ *P*-val
LPD1	8	3	0.0588	13	4	0.0851	0	3	0.235
LPD2	4	8	0.827	3	3	1	0	2	0.467
LPD3	9	67	*1.45* × *10*^*−08*^	5	15	*0.00977*	4	17	*0.00299*
LPD4	14	21	1	14	15	0.619	1	2	0.987
LPD5	65	5	*2.20* × *10*^*−16*^	19	1	*0.000180*	34	0	*1.15* × *10*^*−11*^
LPD6	13	22	0.802	5	5	1	2	4	0.657
DESNT	13	66	*1.17* × *10*^*−06*^	6	15	*0.0207*	9	24	*0.00274*
LPD8	9	6	0.193	8	4	0.540	4	1	0.371

	*PTEN*			*SPOP*			*CHD1*		
	Non-homdel	Homdel	*χ*^2^ *P*-val	Non-mut	Mut	*χ*2 *P*-val	Non-homdel	Homdel	*χ*^2^ *P*-val
LPD1	10	1	0.896	8	3	0.213	9	2	0.309
LPD2	12	0	0.284	12	0	0.436	12	0	0.756
LPD3	55	21	*0.000894*	73	3	*0.0400*	76	0	*0.0211*
LPD4	35	0	*0.0174*	31	4	1	34	1	0.603
LPD5	67	3	*0.00830*	51	19	*4.46* × *10*^*−06*^	57	13	*7.69* × *10*^*−06*^
LPD6	29	6	0.903	32	3	0.825	34	1	0.603
DESNT	60	19	*0.0167*	75	4	0.0795	76	3	0.432
LPD8	15	0	0.195	14	1	0.889	14	1	1

Statistically significant differences are italicised.

### Altered patterns of gene expression and DNA methylation

We examined samples assigned to each OAS-LPD signature for genes with significantly altered expression levels in all eight datasets (*P* < 0.05 after FDR correction, samples in the LPD group vs all other LPD categories from the same dataset, Supplementary data 1). LPD3 cancer samples exhibited seven commonly overexpressed genes, including ERG, GHR and HDAC1. Pathway analysis suggested the involvement of Stat3 gene signalling (Fig. S6a, Supplementary data 2). LPD5 exhibited 47 significantly overexpressed genes and 13 under-expressed genes. Many of the genes had established roles in fatty acid metabolism and the control of secretion (Fig. S6b). LPD6- and LPD8 cancers had failed to exhibit statistically significant changes in genetic alteration or clinical outcome in this study, but did have characteristic altered patterns of gene expression (Fig. S6c, e). The five genes commonly overexpressed in LPD6 cancers suggested involvement in metal ion homoeostasis. In total, 30 genes were overexpressed, and 36 genes under-expressed in in LPD8 cancers, including several genes involved in extracellular matrix organisation. Cross- referencing differential methylation data available for the TCGA dataset with genes associated with each LPD group indicated that many expression changes may be explained, at least in part, by changes in DNA methylation (Fig. 5, Fig. S7, Supplementary data 3).

### DESNT as a signature of metastasis

The MSKCC study includes data from 19 metastatic cancer samples. For each metastatic sample, DESNT was the most abundant signature when OAS-LPD was applied (Fig. 3d). Two of the studied datasets (MSKCC and Erho) had publicly available annotations, indicating that the patients, from which primary cancer expression profiles were examined, had progressed to develop metastasis after prostatectomy (Fig. S3). From nine cancer patients developing metastasis in the MSKCC dataset, five occurred from samples in which the DESNT signature is most common (*X*^2^ test, *P* = 0.0017), and of 212 cancer patients developing metastases in the Erho dataset, 50 were from DESNT cancers (*X*^2^ test, *P* = 0.0019) (Fig. S8). From these studies, we concluded that DESNT cancers have an increased risk of developing metastasis, consistent with the higher risk of PSA failure. For the Erho dataset, membership of LPD1 was associated with lower risk of metastasis (*X*^2^ test, *P* = 0.026, Fig. S8).

To further investigate the underlying nature of DESNT cancer, we used the transcriptome profile for each primary prostate cancer sample to investigate associations with the 17,697 signatures and pathways annotated in the MSigDB database. The top 20 signatures, where expression was associated with the proportion of DESNT, are shown in Table S2. The third most significant correlation was to genes downregulated in metastatic prostate cancer. This resulting data give additional clues to the underlying biology of DESNT cancer, including associations with genes altered in ductal breast cancer, in stem cells and during FGFR1 signalling.

## Discussion

We have confirmed a key prediction of the DESNT cancer model by demonstrating that the presence of a small proportion of the DESNT cancer signature confers poorer outcome. The proportion of DESNT signature can be considered a continuous variable, such that as DESNT cancer content increases, the outcome became worse. This observation led to the development of nomograms for estimating PSA failure at 3, 5 and 7 years following prostatectomy. The result provides an extension of previous studies in which nomograms incorporating Gleason score, stage and PSA value have been used to predict outcome following surgery.^25^

The match between the eight underlying signatures detected for the MSKCC and CancerMap datasets was used as the basis for developing a novel classification framework for prostate cancer. A new algorithm called OAS-LPD was developed to allow rapid assessment of the presence of the signatures in individual cancer samples. In total, four clinically or genetically distinct subgroups were identified (DESNT, LPD3, LPD4 and LPD5, Fig. 5). The functional significance of the new disease groupings, for example, in determining drug sensitivity, remains to be established. However, with the use of OAS-LPD, it will be possible to undertake assessments of the response of patients in each of the groups DESNT, LPD3, LPD and LPD5 to drug treatments. There is limited overlap between the new classification and previously proposed subgroups based on genetic alterations.^16,26–29^

Multiplatform data (expression, mutation and methylation data from each cancer sample) are available for many cancer types, for example from The Cancer Genome Atlas. This has prompted the development of additional methods for sub-class discovery that can combine information from different platforms, including the copula-mixed model,^30^ Bayesian consensus clustering,^31^ and the iCluster model.^16^ Such approaches can suffer from the problem of sample assignment to a particular cluster or group, and the failure to take into consideration the heterogeneous composition of individual cancer samples. These observations highlight the need to develop methods similar to LPD that can be applied to multiplatform data.

An important issue for patients diagnosed with prostate cancer is that the clinical outcome is highly variable, and precise prediction of the course of disease progression at the time of diagnosis is not possible.^32^ In some studies, the use of population PSA screening can reduce mortality from prostate cancer by up to 21%.^33^ However many, if not most, prostate cancers that are currently detected by PSA screening are clinically insignificant.^34^ Overdiagnosis of clinically insignificant prostate cancer is a major issue, and is set to increase still further.^35^ There is therefore an urgent need for the identification of cancer categories that are associated with clinically aggressive or indolent disease to allow the targeting of radical therapies to men that need them. For breast cancer, unsupervised hierarchical clustering of transcriptome data resulted in a classification system that is routinely used to guide the management and treatment of this disease. Here we established a novel classification framework for the analysis of prostate cancer that has its origins in unsupervised analyses of transcriptome data. In future studies, we plan to analyse the utility of DESNT and other LPD processes (particularly LPD3, LPD4 and LPD5) in managing prostate cancer patients, including predicting the response to drug treatment. This will be performed through the assessment of LPD status in the contexts of established clinical trials. For evaluation, we would plan to use each LPD assignment (e.g. DESNT, LPD3, LPD4 and LPD5) as a continuous variable, as illustrated here by the development of a nomogram for the use of DESNT in predicting PSA failure. In conclusion, our results highlight the importance of devising and using more sophisticated approaches for the analysis of genomic datasets from all biological systems.

## Supplementary information

Supplementary Material

Data Set 1

Data Set 2

Data Set 3

## Data Availability

The datasets analysed during this study are available (Table 1). The majority are available from the Gene Expression Omnibus repository: MSKCC:^7^ https://www.ncbi.nlm.nih.gov/geo/query/acc.cgi?acc=GSE21034 CancerMap:^14^ https://www.ncbi.nlm.nih.gov/geo/query/acc.cgi?acc=GSE94767 Klein:^17^ https://www.ncbi.nlm.nih.gov/geo/query/acc.cgi?acc=GSE62667 CamCap:^6^ https://www.ncbi.nlm.nih.gov/geo/query/acc.cgi?acc=GSE70768 and https://www.ncbi.nlm.nih.gov/geo/query/acc.cgi?acc=GSE70769 Erho:^18^ https://www.ncbi.nlm.nih.gov/geo/query/acc.cgi?acc=GSE46691 Karnes:^19^ https://www.ncbi.nlm.nih.gov/geo/query/acc.cgi?acc=GSE62116 Stephenson:^15^ data available from the corresponding author of this paper. TCGA:^16^ data available from the TCGA Data Portal https://portal.gdc.cancer.gov/projects/TCGA-PRAD.
