# Supplementary material for: A novel stratification framework for predicting outcome in patients with prostate cancer
Source: Br J Cancer. 2020 Mar 20;122(10):1467–76. doi: 10.1038/s41416-020-0799-5 (PMC7217762; doi:10.1038/s41416-020-0799-5)
Supplement: Supplementary file 1 — Supplementary Material [file 41416_2020_799_MOESM1_ESM.docx]

**Supplementary Material**

**Availability of data and material**

The datasets analysed during the current study are available (table 2 main text). The majority are available from the Gene Expression Omnibus repository:

- MSKCC^1^ : https://www.ncbi.nlm.nih.gov/geo/query/acc.cgi?acc=GSE21034
- CancerMap^2^ : ﻿ https://www.ncbi.nlm.nih.gov/geo/query/acc.cgi?acc=GSE94767
- Klein^3^ : https://www.ncbi.nlm.nih.gov/geo/query/acc.cgi?acc=GSE62667
- CamCap^4^ : https://www.ncbi.nlm.nih.gov/geo/query/acc.cgi?acc=GSE70768 and https://www.ncbi.nlm.nih.gov/geo/query/acc.cgi?acc=GSE70769
- Erho^5^ : https://www.ncbi.nlm.nih.gov/geo/query/acc.cgi?acc=GSE46691
- Karnes^6^ : https://www.ncbi.nlm.nih.gov/geo/query/acc.cgi?acc=GSE62116
- Stephenson^7^ : Data available from the corresponding author of this paper.
- TCGA^8^: Data available from the TCGA Data Portal <https://portal.gdc.cancer.gov/projects/TCGA-PRAD>

**References**

1 Taylor BS, Schultz N, Hieronymus H, *et al.* Integrative Genomic Profiling of Human Prostate Cancer. *Cancer Cell* 2010; **18**: 11–22.

2 Luca B, Brewer DS, Edwards DR, *et al.* DESNT: A Poor Prognosis Category of Human Prostate Cancer. *Eur Urol Focus* 2018; **4**: 842–50.

3 Klein EA, Yousefi K, Haddad Z, *et al.* A genomic classifier improves prediction of metastatic disease within 5 years after surgery in node-negative high-risk prostate cancer patients managed by radical prostatectomy without adjuvant therapy. *Eur Urol* 2015; **67**: 778–86.

4 Ross-Adams H, Lamb ADD, Dunning MJJ, *et al.* Integration of copy number and transcriptomics provides risk stratification in prostate cancer: A discovery and validation cohort study. *EBioMedicine* 2015; **2**: 1133–44.

5 Erho N, Crisan A, Vergara IA, *et al.* Discovery and validation of a prostate cancer genomic classifier that predicts early metastasis following radical prostatectomy. *PLoS One* 2013; **8**: e66855.

6 Karnes RJ, Bergstralh EJ, Davicioni E, *et al.* Validation of a Genomic Classifier that Predicts Metastasis Following Radical Prostatectomy in an At Risk Patient Population. *J Urol* 2013; **190**: 2047–53.

7 Stephenson AJ, Smith A, Kattan MW, *et al.* Integration of gene expression profiling and clinical variables to predict prostate carcinoma recurrence after radical prostatectomy. *Cancer* 2005; **104**: 290–8.

8 Network CGAR, Cancer Genome Atlas Research Network. The Molecular Taxonomy of Primary Prostate Cancer. *Cell* 2015; **163**: 1011–25.

**Supplementary Tables and Figures**

|  | TCGA | | | CancerMap | | | | CamCap | | | |
| --- | --- | --- | --- | --- | --- | --- | --- | --- | --- | --- | --- |
|  | Benign | Primary | χ^2^ *P*-val | Benign | Primary | | χ^2^ *P*-val | Benign | Primary | χ^2^ *P*-val | |
| LPD1 | 0 | 11 | 0.466092 | 9 | 13 | 0.195522 | | 2 | 7 | | 1 |
| LPD2 | 15 | 12 | 7.89E-13 | 4 | 3 | 0.165632 | | 17 | 4 | | 1.21E-08 |
| LPD3 | 1 | 76 | 0.00335 | 0 | 22 | 0.004958 | | 0 | 36 | | 0.000302 |
| LPD4 | 11 | 35 | 0.00957 | 16 | 23 | 0.044844 | | 30 | 5 | | 5.02E-17 |
| LPD5 | 0 | 70 | 0.001781 | 1 | 24 | 0.010098 | | 0 | 71 | | 1.75E-08 |
| LPD6 | 1 | 35 | 0.149512 | 5 | 7 | 0.404231 | | 6 | 19 | | 0.993199 |
| LPD7 | 0 | 79 | 0.000687 | 1 | 24 | 0.010098 | | 0 | 57 | | 1.20E-06 |
| LPD8 | 15 | 15 | 3.60E-11 | 11 | 10 | 0.012093 | | 18 | 8 | | 4.94E-07 |
|  | *MSKCC* | | | *Glinsky* | | | |  | | | |
|  | Benign | Primary | χ^2^ *P*-val | Benign | Primary | χ^2^ *P*-val | |  | | | |
| LPD1 | 3 | 18 | 0.852347 | - | - | - | |  |  |  |  |
| LPD2 | 12 | 3 | 6.30E-10 | 3 | 4 | 0.050471 | |  |  |  |  |
| LPD3 | 0 | 34 | 0.004501 | 0 | 18 | 0.166692 | |  |  |  |  |
| LPD4 | 6 | 19 | 0.584004 | 1 | 10 | 1 | |  |  |  |  |
| LPD5 | 0 | 22 | 0.037682 | 0 | 19 | 0.146293 | |  |  |  |  |
| LPD6 | 0 | 11 | 0.225693 | 0 | 4 | 1 | |  |  |  |  |
| LPD7 | 0 | 19 | 0.061832 | 0 | 14 | 0.276438 | |  |  |  |  |
| LPD8 | 8 | 5 | 0.000112 | 7 | 9 | 0.000149 | |  |  |  |  |

**Supplementary Table 1.** The distribution of non-cancerous (benign) prostate samples amongst LPD subgroups.

| **Pathway** | **Pearson's R squared** | **Pubmed ID** | **Description** |
| --- | --- | --- | --- |
| TURASHVILI BREAST DUCTAL CARCINOMA VS DUCTAL NORMAL DN | -0.683105732 | [17389037](http://www.ncbi.nlm.nih.gov/entrez/query.fcgi?CMD=search&CrntRpt=DocSum&DB=pubmed&cmd=search&db=pubmed&term=17389037) | Genes down-regulated in ductal carcinoma vs normal ductal breast cells. |
| TURASHVILI BREAST DUCTAL CARCINOMA VS LOBULAR NORMAL DN | -0.680108244 | [17389037](http://www.ncbi.nlm.nih.gov/entrez/query.fcgi?CMD=search&CrntRpt=DocSum&DB=pubmed&cmd=search&db=pubmed&term=17389037) | Genes down-regulated in ductal carcinoma vs normal lobular breast cells. |
| CHANDRAN METASTASIS DN | -0.676822998 | [17430594](http://www.ncbi.nlm.nih.gov/entrez/query.fcgi?CMD=search&CrntRpt=DocSum&DB=pubmed&cmd=search&db=pubmed&term=17430594) | Genes down-regulated in metastatic tumors from the whole panel of patients with prostate cancer. |
| DELYS THYROID CANCER DN | -0.672689295 | [17621275](http://www.ncbi.nlm.nih.gov/entrez/query.fcgi?CMD=search&CrntRpt=DocSum&DB=pubmed&cmd=search&db=pubmed&term=17621275) | Genes down-regulated in papillary thyroid carcinoma (PTC) compared to normal tissue. |
| BMI1 DN.V1 DN | -0.67215877 | [17452456](http://www.ncbi.nlm.nih.gov/entrez/query.fcgi?CMD=search&CrntRpt=DocSum&DB=pubmed&cmd=search&db=pubmed&term=17452456) | Genes down-regulated in DAOY cells (medulloblastoma) upon knockdown of BMI1 gene by RNAi. |
| TURASHVILI BREAST LOBULAR CARCINOMA VS DUCTAL NORMAL DN | -0.666577782 | [17389037](http://www.ncbi.nlm.nih.gov/entrez/query.fcgi?CMD=search&CrntRpt=DocSum&DB=pubmed&cmd=search&db=pubmed&term=17389037) | Genes down-regulated in lobular carcinoma vs normal ductal breast cells. |
| CSR LATE UP.V1 DN | -0.654391638 | [14737219](http://www.ncbi.nlm.nih.gov/entrez/query.fcgi?CMD=search&CrntRpt=DocSum&DB=pubmed&cmd=search&db=pubmed&term=14737219) | Genes down-regulated in late serum response of CRL 2091 cells (foreskin fibroblasts). |
| LEE NEURAL CREST STEM CELL DN | -0.649845872 | [18037878](http://www.ncbi.nlm.nih.gov/entrez/query.fcgi?CMD=search&CrntRpt=DocSum&DB=pubmed&cmd=search&db=pubmed&term=18037878) | Genes down-regulated in the neural crest stem cells (NCS), defined as p75+/HNK1+ [GeneID=4804;27087]. |
| VECCHI GASTRIC CANCER EARLY DN | -0.64509729 | [17297478](http://www.ncbi.nlm.nih.gov/entrez/query.fcgi?CMD=search&CrntRpt=DocSum&DB=pubmed&cmd=search&db=pubmed&term=17297478) | Down-regulated genes distinguishing between early gastric cancer (EGC) and normal tissue samples. |
| GSE25088 WT VS STAT6 KO MACROPHAGE ROSIGLITAZONE AND IL4 STIM DN | -0.644420534 | [21093321](http://www.ncbi.nlm.nih.gov/entrez/query.fcgi?CMD=search&CrntRpt=DocSum&DB=pubmed&cmd=search&db=pubmed&term=21093321) | Genes down-regulated in bone marrow-derived macrophages treated with IL4 [GeneID=3565] and rosiglitazone [PubChem=77999]: wildtype versus STAT6 [GeneID=6778] knockout. |
| WU SILENCED BY METHYLATION IN BLADDER CANCER | -0.644402585 | [17456585](http://www.ncbi.nlm.nih.gov/entrez/query.fcgi?CMD=search&CrntRpt=DocSum&DB=pubmed&cmd=search&db=pubmed&term=17456585) | Genes silenced by DNA methylation in bladder cancer cell lines. |
| ACEVEDO FGFR1 TARGETS IN PROSTATE CANCER MODEL DN | -0.64107159 | [18068632](http://www.ncbi.nlm.nih.gov/entrez/query.fcgi?CMD=search&CrntRpt=DocSum&DB=pubmed&cmd=search&db=pubmed&term=18068632) | Genes down-regulated during prostate cancer progression in the JOCK1 model due to inducible activation of FGFR1 [GeneID=2260] gene in prostate. |
| CORRE MULTIPLE MYELOMA DN | -0.635300151 | [17344918](http://www.ncbi.nlm.nih.gov/entrez/query.fcgi?CMD=search&CrntRpt=DocSum&DB=pubmed&cmd=search&db=pubmed&term=17344918) | Genes down-regulated in multiple myeloma (MM) bone marrow mesenchymal stem cells. |
| PEPPER CHRONIC LYMPHOCYTIC LEUKEMIA UP | -0.633518278 | [17287849](http://www.ncbi.nlm.nih.gov/entrez/query.fcgi?CMD=search&CrntRpt=DocSum&DB=pubmed&cmd=search&db=pubmed&term=17287849) | Genes up-regulated in CD38+ [GeneID=952] CLL (chronic lymphocytic leukemia) cells. |
| POOLA INVASIVE BREAST CANCER DN | -0.630569526 | [15864312](http://www.ncbi.nlm.nih.gov/entrez/query.fcgi?CMD=search&CrntRpt=DocSum&DB=pubmed&cmd=search&db=pubmed&term=15864312) | Genes down-regulated in atypical ductal hyperplastic tissues from patients with (ADHC) breast cancer vs those without the cancer (ADH). |
| GSE3982 NKCELL VS TH1 UP | -0.630227356 | [16474395](http://www.ncbi.nlm.nih.gov/entrez/query.fcgi?CMD=search&CrntRpt=DocSum&DB=pubmed&cmd=search&db=pubmed&term=16474395) | Genes up-regulated in comparison of NK cells versus Th1 cells. |
| GO MONOCYTE DIFFERENTIATION | -0.629962124 | NA | The process in which a relatively unspecialized myeloid precursor cell acquires the specialized features of a monocyte. |
| LIU PROSTATE CANCER DN | -0.629526171 | [16618720](http://www.ncbi.nlm.nih.gov/entrez/query.fcgi?CMD=search&CrntRpt=DocSum&DB=pubmed&cmd=search&db=pubmed&term=16618720) | Genes down-regulated in prostate cancer samples. |
| OSADA ASCL1 TARGETS DN | -0.625032708 | [18339843](http://www.ncbi.nlm.nih.gov/entrez/query.fcgi?CMD=search&CrntRpt=DocSum&DB=pubmed&cmd=search&db=pubmed&term=18339843) | Genes down-regulated in A549 cells (lung cancer) upon expression of ASCL1 [GeneID=429] off a viral vector. |
| GAUSSMANN MLL AF4 FUSION TARGETS F UP | -0.623309469 | [17130830](http://www.ncbi.nlm.nih.gov/entrez/query.fcgi?CMD=search&CrntRpt=DocSum&DB=pubmed&cmd=search&db=pubmed&term=17130830) | Up-regualted genes from the set F (Fig. 5a): specific signature shared by cells expressing AF4-MLL [GeneID=4299;4297] alone and those expressing both AF4-MLL and MLL-AF4 fusion proteins. |

**Supplementary Table 2.** Top 20 correlations between MSigDB database signature status and DESNT content. The transcriptome profile for each prostate cancer was used to calculate the status of the 17,697 signatures and pathways annotated in the MSigDB database. The top 20 correlations to DESNT Gamma are shown.

**Supplementary Figure 1.** Cox Model for DESNT cancers assessed by LPD. **(a)** graphical representation of HR for each covariate and 95% confidence intervals of HR. **(b)** HR, 95% CI and Wald test statistics of the Cox model. **(c)** Calibration plots for the internal validation of the nomogram, using 1000 bootstrap resamples. Solid black line represents the apparent performance of the nomogram, blue line the bias-corrected performance and dotted line the ideal performance. **(d)** Calibration plots for the external validation of the nomogram using the CamCap dataset. Solid line corresponds to the observed performance and dotted line to the ideal performance.

**Supplementary Figure 2.** Correlation in expression profiles between MSKCC and CancerMap LPD groups. Correlations of the average levels of gene expression for cancers assigned to each LPD group are presented. The expression levels of each gene have been normalised across all samples to mean 0 and standard deviation 1. Even for the lower Pearson Coefficients the correlation is highly statistically significant (Pearson's product-moment correlation test).

**Supplementary Figure 3.** Add One Sample Latent Process Decomposition (OAS-LPD) for eight prostate cancer transcriptome datasets. See Figure 1 for a description of the plots with the exception that in this Figure the different colours denote different Gleason Sums. Vertical axis is the fraction of the sample (Gamma).

**Supplementary Figure 4.** Cox Model for DESNT cancers assessed by OAS-LPD. **(a)** graphical representation of HR for each covariate and 95% confidence intervals of HR. **(b)** HR, 95% CI and Wald test statistics of the Cox model. **(c)** Calibration plots for the internal validation of the nomogram, using 1000 bootstrap resamples. Solid black line represents the apparent performance of the nomogram, blue line the bias-corrected performance and dotted line the ideal performance. **(d)** Calibration plots for the external validation of the nomogram using the CamCap dataset. Solid line corresponds to the observed performace and dotted line to the ideal performance.

**Supplementary Figure 5.** Nomogram model developed to predict PSA free survival at 1, 3, 5 and 7 years for DESNT cancer assessed by OAS-LPD. Assessing a single patient each clinical variable has a corresponding point score (top scales). The point scores for each variable are added to produce a total points score for each patient. The predicted probability of PSA free survival at 1, 3, 5 and 7 years can be determined by drawing a vertical line from the total points score to the probability scales below.


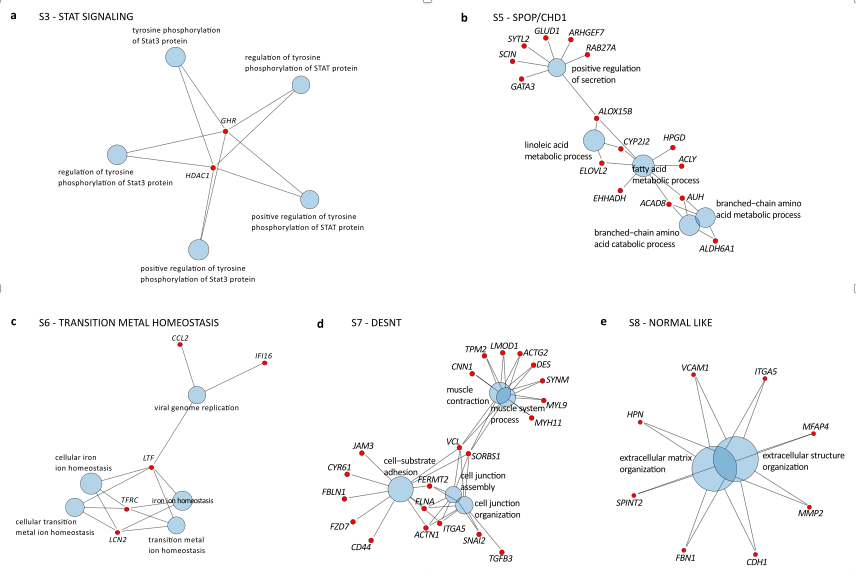


**Supplementary Figure 6.** GO pathway over-representation analysis for the lists of differentially expressed genes in each process. For each gene set, up to 5 pathways with the lowest p-values are represented. Blue nodes correspond to pathways, red nodes to genes, and the vertices indicate the involvement of the gene in the pathway. The size of blue nodes is inversely proportional to the over-representation p-value.

**Supplementary Figure 7**. Methylation heatmap of the differentially methylated genes in each process (see Methods). Columns correspond to samples and rows to probes mapping to differentially methylated CpG clusters for each gene. Probes are in the same order as in Supplementary Data 3. Red colour corresponds to hyper-methylated probes, whereas the green colour corresponds to hypo-methylated probes. CL1-CL4 are the methylation subgroups published as part of The Cancer Genome Atlas Project.

**Supplementary Figure 8.** Correlation of metastatic cancer with OAS-LPD category. (**a)** OAS-LPD assignments were determined based on analysis of expression profiles of primary cancers as shown in Fig. S2. The frequency of cancers associated with developing metastases in each LPD category is shown for the Erho *et al*^5^ (upper panel) and MSKCC^1^ (lower panel) datasets. (**b**) Signature assignment gamma values for the 19 metastases reported as part of the MSKCC dataset were subject to OAS-LPD. In all cases LPD7 (DESNT) was the dominant expression signature detected.

**Legends for Supplementary data**

**Supplementary data 1. S**amples assigned to each OAS-LPD signature for genes with significantly altered expression levels in all eight datasets (P < 0.05 after FDR correction, samples in LPD group vs all other LPD categories from the same dataset).

**Supplementary data 2.** Gene Ontology enrichment analysis using genes with altered expression in each LPD groups.

**Supplementary data 3.** Differential methylation. Consistently over and under expressed genes for each LPD group (Supplementary Data 1) were assesses for differential methylation as described in the Materials and Methods. Differentially methylated loci are listed.

**ANALYSIS OUTLINE**

**Datasets**

MSKCC

(*n* = 160)

CancerMap

(*n* = 154)

CamCap

(*n* = 220*)*

Klein

(*n* = 182)

TCGA

(*n* = 376)

Erho

(*n* = 545)

Karnes

(*n* = 232)

Stephenson

(*n* = 89)

Combat+quantile normalisation

Processed

MSKCC

(*n* = 160)

Processed

CancerMap

(*n* = 154)

Processed

CamCap

(*n* = 220*)*

Processed Klein

(*n* = 182)

Processed TCGA

(*n* = 376)

Processed Erho

(*n* = 545)

Processed Karnes

(*n* = 232)

Processed

Stephenson

(*n* = 89)

**Combined Datasets used for Survival Analysis (Fig. 2a,b)**

LPD Combined Survival (*n* = 503)

MSKCC

(*n* = 160)

CancerMap

(*n* = 154)

CamCap

(*n* = 220*)*

Stephenson

(*n* = 89)

Tumour tissue from prostate

(*n* = 131)

Tumour tissue from prostate

(*n* = 137)

Tumour tissue from prostate

(*n* = 78)

Tumour tissue from prostate

(*n* = 147)

**Training Dataset for Construction of Nomogram (Fig. 2c)**

LPD Combined Survival train (*n* = 318)

MSKCC

(*n* = 160)

CancerMap

(*n* = 154)

Stephenson

(*n* = 89)

Tumour tissue from prostate

(*n* = 131)

Tumour tissue from prostate

(*n* = 137)

Tumour tissue from prostate

(*n* = 78)

**LPD**

LPD was run on four datasets to produce four process models and four sets of gamma values.

MSKCC (*n* = 160)

CancerMap (*n* = 154)

CamCap (*n* = 220)

Stephenson (*n* = 78)

MSKCC LPD model

*LPD_γ_i,j,MSKCC_*

CancerMap LPD model

*LPD_γ_i,j,CancerMap_*

Stephenson LPD model

*LPD_γ_i,j,Stephenson_*

CamCap LPD model

*LPD_γ_i,j,CamCap_*

Where LPD_γ_i,j,D_ is the gamma value assigned to sample *i* for process *j* in dataset *D*

**OAS LPD**

Dataset (*D*)

OAS -LPD

MSKCC LPD model

*γ_i,j,D_*

For each of the eight datasets (*D* in MSKCC, CancerMap, Stephenson, CamCap, Klein, TCGA, Erho, Karnes, and 19 metastatic samples from MSKCC paper) the MSKCC LPD model is applied to obtain gamma value assigned to sample *i* for process *j* in dataset *D*, γ_i,j,D_.

**DESNT as continuous signature predicts survival**

For *D* in MSKCC, CancerMap, Stephenson, and CamCap (label: LPD_combined_survival) a Cox model with BCR as outcome was applied with LPD_γ_i,j,D_.

LPD combined survival (*n* = 503)

Cox regression

LPD_*γ_i,DESNT_*

Significance and HR

**Nomogram**

For *D* in MSKCC, CancerMap, and Stephenson (label: LPD_combined_survival_train) a Cox model with BCR as outcome was applied with LPD_γ_i,j,D_ and clinical covariates. CamCap was used as a validation set, along with internal validation through bootstrap.

LPD combined survival train (*n* = 318)

Cox regression

LPD_*γ_i,DESNT_*

Significance and HR

Path Stage

Surgical Margins

Gleason

PSA

1000 x bootstrap resample

CamCap (*n* = 185)

validation

**Correlation between MSKCC and CancerMap**

For both MSKCC and CancerMap there are 8 processes detected (*j* in 1..8). For each sample *i* a characteristic process is assigned based on the maximum gamma i.e.

$${LPD\_A}_{i,D}=\arg\max_{j\in1\ldots8} {LPD\_\gamma}_{i,j,D}$$

Pearson correlations between the expression profiles between the two datasets for each process, *j* was calculated as follows:

1. for each gene, *g*, we select one corresponding probe at random;
2. for each probe we transformed its distribution across all samples to a standard normal distribution;

the mean expression for each gene, $\overline{x_{g,j,D}}$ across the samples assigned to each process (gene subgroup mean i.e. *i* in ${LPD\_A}_{i,D}==j$) and each dataset was determined;

1. the Pearson’s correlation between the subgroup x in dataset A and subgroup y in dataset B is calculated by using the gene subgroup mean in x vs gene subgroup mean in y for all genes i.e. $\overline{x_{g,j,MSKCC}} vs \overline{x_{g,j,CancerMap}}$ for all *g.*

**OAS-LPS confirmation of nomogram**

For *D* in MSKCC, CancerMap, and Stephenson (label: LPD_combined_survival_train) a Cox model with BCR as outcome was applied with γ_i,j,D_ (derived from LPD-OAS) and clinical covariates. CamCap was used as a validation set, along with internal validation through bootstrap.

LPD combined survival train (*n* = 318)

Cox regression

*γ_i,DESNT_*

Significance and HR

Path Stage

Surgical Margins

Gleason

PSA

1000 x bootstrap resample

CamCap (*n* = 185)

validation

**New categories**

For each sample *i* a characteristic process is assigned based on the maximum gamma i.e.

$$A_{i,D}=\arg\max_{j\in1\ldots8} \gamma_{i,j,D}$$

For sample *i*, process *j* and dataset *D*.

**New categories - survival**

For each *j* (process), we calculate whether there is a significant effect on time to biochemical recurrence.

LPD combined survival (*n* = 503)

Log rank test

*A_i_ == j* vs *A_i_ =! j*

Significance

For *j* = LPD3 and *D* in {CancerMap,CamCap,TCGA} we determined whether having ETS+ aberration had an association with survival.

TCGA (*n* = 333)

Log rank test

*ETS* +ve vs *ETS* -ve

Significance

CancerMap (*n* = 137)

Log rank test

*ETS* +ve vs *ETS* -ve

Significance

CamCap (*n* = 147)

Log rank test

*ETS* +ve vs *ETS* -ve

Significance

**New categories - Genetics Alterations**

For *D* = TCGA, each genetic alteration, *a*, and each process, *j*, we calculated if there was a significant over or under representation using a χ^2^-test.

TCGA (*n* = 333)

χ^2^-test

*A_i_ == j* vs *A_i_ =! J*

*a_i_ +ve* vs *a_i_ -ve*

Significance

For *a* = “ETS” we also performed this test in the Cancermap and CamCap datasets.

**New categories - Expression**

MSKCC (*n* = 131)

Moderated *t*-test

*A_i_ == j* vs *A_i_ =! j*

Significance Genes (SigGenes_MSKCC,j_)

Karnes (*n* = 232)

Moderated *t*-test

*A_i_ == j* vs *A_i_ =! j*

Significance Genes (SigGenes_Karnes,j_)

…

Across 8 datasets

Intersect

SigGenes_D,j_

Significance Genes (IntersectSigGenes_j_)

For each process, *j,* and dataset (in all 8 datasets), *D*, a list of significant genes is made. The intersect of these significant genes is assigned as the differentially expressed genes assigned to that process.

**New categories - Methylation**

For each process, *j*, and each gene, *g*, in the significant genes list (IntersectSigGenes_j_), we determine whether there is differential methylation using the TCGA dataset.

TCGA (*n* = 333; g in IntersectSigGenes_j_)

methylMix

*A_i_ == j* vs *A_i_ =! j*

Hypo and hypermethylated genes

**Metastatic disease and DESNT**

1. For the 19 metastatic samples in the MSKCC dataset 100% were assigned to DESNT i.e. $A_{i,MSKCC\_mets}=DESNT$ for all *i*.
2. For *D* in {MSKCC, Erho} for each process, *j*, we determined if there was a significant association between membership of a process (found from primary sample) and whether the patient got metastatic disease.

Erho (*n* = 545)

χ^2^-test

*A_i_ == j* vs *A_i_ =! J*

*Mets +ve* vs *Mets -ve*

Significance

MSKCC (*n* = 131)

χ^2^-test

*A_i_ == j* vs *A_i_ =! J*

*Mets +ve* vs *Mets -ve*

Significance

1. Correlation with datasets in MSigDB

Using the complete combined dataset of all 8 datasets, Z-scores were calculated for each sample, *i*, for each geneset, *gs*, in MsigDB. A pearson correlation was performed between each geneset’s Z-scores and the DESNT gamma value.

Complete merged dataset (*n* = 1958)

Pearson Correlation

*Z_i,GS_ vs γ_i,DESNT_* for all *i*

Significance and effect size

**Transfer of LPD Parameters to the OAS-LPD classification**.

The OAS-LPD classification procedure is made up of two stages:

• The use of standard LPD algorithm on a training set of samples to learn the reference (or model) parameters;

• The use of a modified procedure, specific to OAS-LPD model, to classify a new sample or a set of new samples.  The modified procedure uses the reference parameters derived in step 1.

Stage 1 is identical to a standard LPD learning procedure on a given set of A samples, G genes (which can be 500 or other number) and K processes. Once the stage 1 is finished, the sets of variables (µ_gk_, σ^2^_gk_, and α in Rogers *et al*.2005^1^) are saved and stored for use in stage 2.

$$\mu{}_{gk}=\frac{\sum_{a} Q_{kga}e{}_{ga}}{\sum_{a} Q_{kga}}$$

$$\sigma_{gk}^{2}=\frac{\sum_{a} Q_{kga}\left( e_{ga}-\mu{}_{gk} \right)^{2}}{\sum_{a} Q_{kga}}$$

$$\gamma_{ak}=\alpha_{k}+\sum_{g} Q_{kga}$$

The index *a* ranges *a=1,..,A* over the *A* samples in the data, *g* ranges *g=1,..,G*, over the genes. *k* (or *j*) ranges *k=1,..,K* over the *K* processes (soft clusters) present in the data. $e_{ga}$ encodes the gene expression data values for gene *g* in sample *a*.

In Stage 2, in order to classify a new set of A’ samples, where A’ can be 1 or more patient samples that is/are undergoing classification, the following steps can be followed:

1.     A new instance of the OAS-LPD model is created, using A’ samples, and the same set of *G*genes and *K*used in stage 1.

2.     The sets of variables are initialised with the values determined at stage 1.

3.     The set of variables are inferred using a suitable learning procedure. One such procedure can as follows:

a.     Initialise the *K* components of vector γ_a_ with random values between 0 and 1, with the constraint that they sum to 1 across the *K* components;

b.     For a number of maxIterations iterations (where maxIterations is a positive natural number), do:

       i.         Using   *µ_gk_* and *σ^2^_gk_* as reference variables, calculate *Q_kga_* as in the following equation

$$Q_{kga}=\frac{N(e_{ga}|k,\mu{}_{gk},\sigma_{gk})exp[\psi(\gamma_{ak})]}{\sum_{j=1}^{K} N(e_{ga}|k,\mu{}_{gj},\sigma_{gj})exp[\psi(\gamma_{aj})]}$$

Where a Gaussian distribution is assumed for the data$N(..)$ and the operator $\psi(..)$ is a digamma function.
      ii.         Calculate *γ_ak_* as in the following equation, using α as provided as the reference variables and *Q_kga_* as calculated at step (b)(i):

$$\gamma_{ak}=\alpha_{k}+\sum_{g} Q_{kga}$$

The above algorithmic process (b) is terminated when a log-likelihood function reaches an approximate plateau.

When the algorithm finishes, for each sample, the analysis provides the weighting for each of the gene signatures i.e. γ_ak_ where γ is provided for each *k* (cancer gene signature) of each *a* (sample expression profile). γ encodes the OAS-LPD classification of each *A* sample. γ gives the ideal weighted combination of the gene signatures to replicate the sample expression profile.

^1^Rogers S, Girolami M, Campbell C, Breitling R (2005) The latent process decomposition of cDNA microarray data sets. IEEE/ACM Trans Comput Biol Bioinforma 2: 143–156, doi:10.1109/TCBB.2005.29.
